# Detection and Distinction of Mild Brain Injury Effects in a Ferret Model Using Diffusion Tensor MRI (DTI) and DTI-Driven Tensor-Based Morphometry (D-TBM)

**DOI:** 10.3389/fnins.2018.00573

**Published:** 2018-08-17

**Authors:** Elizabeth B. Hutchinson, Susan C. Schwerin, Kryslaine L. Radomski, Neda Sadeghi, Michal E. Komlosh, M. O. Irfanoglu, Sharon L. Juliano, Carlo Pierpaoli

**Affiliations:** ^1^Section on Quantitative Medical Imaging, National Institute of Biomedical Imaging and Bioengineering, National Institutes of Health, Bethesda, MD, United States; ^2^The Henry M. Jackson Foundation for the Advancement of Military Medicine, Inc., Bethesda, MD, United States; ^3^Department of Anatomy, Physiology, and Genetics, Uniformed Services University of the Health Sciences, Bethesda, MD, United States; ^4^Section on Quantitative Imaging and Tissue Sciences, Eunice Kennedy Shriver National Institute of Child Health and Human Development, National Institutes of Health, Bethesda, MD, United States

**Keywords:** diffusion tensor MRI, ferret, controlled cortical impact, mild traumatic brain injury, tensor-based morphometry, white matter

## Abstract

Mild traumatic brain injury (mTBI) is highly prevalent but lacks both research tools with adequate sensitivity to detect cellular alterations that accompany mild injury and pre-clinical models that are able to robustly mimic hallmark features of human TBI. To address these related challenges, high-resolution diffusion tensor MRI (DTI) analysis was performed in a model of mild TBI in the ferret – a species that, unlike rodents, share with humans a gyrencephalic cortex and high white matter (WM) volume. A set of DTI image analysis tools were optimized and implemented to explore key features of DTI alterations in *ex vivo* adult male ferret brains (*n* = 26), evaluated 1 day to 16 weeks after mild controlled cortical impact (CCI). Using template-based ROI analysis, lesion overlay mapping and DTI-driven tensor-based morphometry (D-TBM) significant differences in DTI and morphometric values were found and their dependence on time after injury evaluated. These observations were also qualitatively compared with immunohistochemistry staining of neurons, astrocytes, and microglia in the same tissue. Focal DTI abnormalities including reduced cortical diffusivity were apparent in 12/13 injured brains with greatest lesion extent found acutely following CCI by ROI overlay maps and reduced WM FA in the chronic period was observed near to the CCI site (ANOVA for FA in focal WM: time after CCI *p* = 0.046, brain hemisphere *p* = 0.0012) often in regions without other prominent MRI abnormalities. Global abnormalities were also detected, especially for WM regions, which demonstrated reduced diffusivity (ANOVA for Trace: time after CCI *p* = 0.007) and atrophy that appeared to become more extensive and bilateral with longer time after injury (ANOVA for D-TBM Log of the Jacobian values: time after CCI *p* = 0.007). The findings of this study extend earlier work in rodent models especially by evaluation of focal WM abnormalities that are not influenced by partial volume effects in the ferret. There is also substantial overlap between DTI and morphometric findings in this model and those from human studies of mTBI implying that the combination of DTI tools with a human-similar model system can provide an advantageous and informative approach for mTBI research.

## Introduction

As the adverse consequences of mild traumatic brain injury (mTBI) become increasingly evident in humans (Carroll et al., 2014; Levin and Diaz-Arrastia, 2015), there is a need to develop human-relevant model systems to study mTBI as well as more sensitive diagnostic markers that can be applied at both the bench and bedside. While conventional non-invasive imaging by CT and MRI are commonly used for moderate and severe TBI (Duhaime et al., 2010), they do not provide robust markers of brain alterations after mTBI and consequently there is urgency to develop quantitative imaging methods better able to detect particular features of neuropathology. Among these methods, diffusion tensor MRI (DTI) has been shown to enable detection of abnormal brain tissue by its sensitivity to the microscale tissue environment, which can be dramatically changed in the presence of cellular alterations and physiologic change. Furthermore, several hallmark pathologies of mTBI such as diffuse axonal injury (DAI) preferentially affect the white matter (WM) of the brain to which DTI anisotropy metrics are highly sensitive (Arfanakis et al., 2002; Shenton et al., 2012; Hulkower et al., 2013). However, to develop DTI markers with the greatest impact we must understand the correspondence of observed DTI abnormalities with meaningful outcomes including their underlying correlates related to cellular structure and biologic function.

Developing and validating advanced MRI tools in experimental model systems has the combined advantages of using identical *in vivo* MRI outcome measures across human and pre-clinical applications as well as the ability to perform fixed tissue analysis by MRI and histology to determine radiologic-pathologic correspondence. While several DTI studies have been performed in models of moderate and severe TBI, only a handful have identified diffusion MRI markers of mild TBI (Bennett et al., 2012; Hylin et al., 2013; Stemper et al., 2015; Yu et al., 2016; Haber et al., 2017). Across these mTBI studies, the most common DTI abnormality is reduced anisotropy in WM, but the timecourse and correlation of decreased FA with histopathology across studies is not consistent. A major caveat is that all existing diffusion MRI studies of mTBI have been performed in rodents, which have little WM compared with gyrencephalic species (Ventura-Antunes et al., 2013). Therefore, adapting mTBI models to species for which WM diffusion can be adequately measured is an important step for identifying the full range of diffusion MRI abnormalities that may indicate cellular and anatomical alterations after mTBI in a sensitive and consistent manner. Histologic evidence from mTBI in swine indicates high levels of DAI pathology despite low severity of the injury (Browne et al., 2011) and recent findings from ferrets demonstrate chronic widespread WM gliosis after mTBI (Schwerin et al., 2017). These observations support the suitability of gyrencephalic species for mTBI experimental models and indicate levels of WM pathology that may be detectable using DTI.

The objective of this study was to evaluate in detail the spatial localization, temporal patterns and quantitative MRI profile of changes following mTBI using high-resolution fixed tissue MRI in brains with human-similar anatomic features. The adult ferret was selected as it is among the smallest mammals with the greatest degree of cortical folding and also has relatively high WM volume. High-resolution *ex vivo* anatomical and diffusion MRI was collected for brains obtained 1 day to 16 weeks after mild controlled cortical impact (CCI) and imaging abnormalities were assessed using a battery of conventional and advanced image analysis tools including region of interest and voxelwise DTI metric comparisons as well as tensor-based morphometry (TBM) to identify local volume changes after TBI. The ability of advanced diffusion MRI methodology to detect and distinguish neuropathology that is invisible to other non-invasive methods was evaluated as well as the suitability of experimental mTBI in the ferret to bridge what has been learned from rodent studies with what has been observed in human TBI research.

## Materials and Methods

### Experimental Design

Whole brain specimens from adult male ferrets were imaged in this study including control specimens (*n* = 9) and brains obtained at the following time points after experimental mTBI: 1 day (*n* = 3), 1 week (*n* = 4), 4 weeks (*n* = 6), and 16 weeks (*n* = 4). Blinding of the researchers was not possible in this study as the ferret data was collected in sequential small groups over several years and the nature of the experiments required researcher familiarity with each animal precluding blinding. DTI from one of the injured brains was included in a previous publication to support *in vivo* findings in the same animal (Hutchinson et al., 2016). An MRI ferret brain template computed from 8 of the 9 controls included in this study is publicly available (Hutchinson et al., 2017b). This template is the source of template-based ROIs and registration targets in the present study. All housing and procedures were approved by the Uniformed Services University Animal Care and Use Committee and all aspects of the animal protocol were designed and implemented according to the institutional and national guidelines.

### Controlled Cortical Impact in the Ferret Brain

Surgical procedures to induce a mild CCI to the left somatosensory cortex (CTX) were performed according to the methods described in Schwerin et al. (2017). Surgical coordinates were pre-determined with reference to the anterior cranial landmark of the junction of the supraorbital crests using a presurgical structural MRI for 3D-rendering and landmark measurements of the brain and skull surfaces with ITKsnap^1^ software. On the day of surgery, ferrets were anesthetized using inhaled isoflurane (4–5% induction and 2–3% maintenance) and intubated for the duration of the surgery. Physiological monitoring for heart rate, respiration and temperature were used to adjust anesthesia and temperature during the procedure. First, a cranial window was placed at the pre-determined skull location using a bone drill. Then, CCI was directed to the left hemisphere somatosensory CTX using an electromagnetically controlled stereotaxic impactor (Impact One Stereotaxic Impactor, Leica Microsystems, Buffalo Grove, IL, United States) with: tip diameter = 3 mm, velocity = 5 m/s, depth = 2 mm, and dwell time = 100 ms. To reduce the risk of infection, all surgical procedures were performed using aseptic technique and in a sterilized operating suite and amoxicillin trihydrate/clavulanate potassium (Clavamox, Zoetis, Kalamazoo, MI, United States) was given orally twice a day for 3–5 days after surgery (1 ml; 62.5 mg/kg). Additionally, the ferrets received two-staged doses of buprenorphine intramuscularly (0.01–0.03 mg/kg) for pain management. Control animals in this study did no undergo any surgical procedures, antibiotics or analgesics.

At the prescribed end time point, ferrets were deeply anesthetized by isoflurane inhalation (5% in oxygen) and an i.p. overdose of Euthasol (50 mg/kg). Upon cessation of reflexes, ferrets were transcardially perfused with 1 L of ice-cold phosphate buffered saline (PBS) (pH 7.4) followed by 1 L of 4% paraformaldehyde solution in PBS (Santa Cruz Biotechnology) containing 47.6 mg of heparin (Sigma-Aldrich). The brains were extracted from the skulls, post-fixed in 4% paraformaldehyde for 8–10 days, and then transferred to a storage solution containing 0.03% sodium azide in PBS. Following at least 1 week of rehydration in this solution, specimens were immersed in Fluorinert (FC-3283, 3M, St. Paul, MN, United States) in a 25-mm glass NMR tube for imaging.

### *Ex vivo* Diffusion-Weighted Image (DWI) Acquisition and Processing

Prepared brain specimens were imaged using a 25-mm linear RF coil and Bruker 7T vertical wide-bore microimaging system (Bruker, Bilirica, MA, United States) with Avance III Spectrometer running Paravision 5.1 software and three GREAT60 gradient amplifiers. For structural MRI and quantitative T2 imaging, a 3D multi-slice multi-echo (MSME) pulse sequence was used with TE/TR = 10–100/3000 ms, nex = 1, reps = 1, and the following spatial parameters: FOV = 26 mm × 40 mm × 20 mm, matrix = 104 × 160 × 80 resulting in isotropic voxel dimensions of 250 microns. Total scan time for this acquisition was 6 h 57 min. T2 maps were generated in the native space by the Carr-Purcell Meibloom Gill multiple TE approach. A single image with TE = 40 ms was used for registration and as an aligned structural target for use with DWI processing. This image was aligned to an *ex vivo* ferret brain template by rigid landmark registration using four midline WM structures.

For DTI, a total of 88 diffusion-weighted image (DWI) volumes were acquired with the same spatial geometry and dimensions as the T2 MRI. A standard 3D EPI pulse sequence was modified slightly to exclude gradient crushers and used with the following imaging parameters: TE/TR = 36/700 ms, nex = 1, 8 segments and two repetitions for each DWI with opposite phase encode directions (to enable subsequent corrections for geometric distortions described in the next section). The DWI sampling scheme was b(in s/mm^2^)/# gradient directions = 100/6, 200/6, 500/6, 1000/6, 1700/32, and 3800/32. The acquisition time for a single image was 7.5 min and the total acquisition time for all DWIs and repetitions was 22 h.

The DWIs from each ferret brain were processed using the TORTOISE3 software package (Irfanoglu et al., 2017) for apparent motion artifacts using rigid alignment of the DWIs and for geometric distortions using the DRBUDDI module of TORTOISE to warp and combine repetitions with opposite phase encode direction with the aligned structural image as a target. The diffusion tensor (DT) was then fit using a non-linear least squares algorithm in the DIFFCALC module of TORTOISE (Pierpaoli et al., 2010) and scalar maps for DTI metrics were generated from the eigenvalues of the DT (λ_1_, λ_2_, and λ_3_) including fractional anisotropy (FA) (Basser and Pierpaoli, 1996), Trace (TR = λ_1_ + λ_2_ + λ_3_), axial diffusivity (AD = λ_1_), and radial diffusivity [RD = (λ_2_ + λ_3_)/2]. Directionally encoded color (DEC) maps were produced using the absolute value scheme (Pajevic and Pierpaoli, 1999).

### Registration Methods: Structural and DTI-Driven Diffeomorphic Registration

For both anatomical MRI and DTI registration as well as ROI masks in template space, existing *ex vivo* ferret brain templates were used.

#### Structural MRI Registration

Structural images of each individual brain volume were spatially coregistered to the *ex vivo* structural template using the “AntsRegistration” command in the ANTs registration software (Avants et al., 2008). First an affine registration to the template was performed followed by a non-linear diffeomorphic registration with cross-correlation similarity metric, symmetric normalization transformation model with 0.25 gradient step size and 140 × 140 × 140 × 100 iterations per level with smoothing sigmas of 0.25 × 0.1 × 0.05 × 0.0. The transformation for each brain volume was applied to warp each quantitative T2 map into template space and also for use with conventional TBM methods described later in this section.

#### DTI Registration

Non-linear registration of individual DT volumes to an existing *ex vivo* DTI template was accomplished using DRTAMAS (Irfanoglu et al., 2016) registration software which makes use of both scalar and tensor-based information and uses an initial affine registration followed by a fully deformable diffeomorphic registration. When applicable, registration settings and parameters for DRTAMAS were identical to those used for structural registration and given above. Individual DT volumes for each brain in template space were then used to generate DTI metric maps for voxelwise analysis and the deformation maps generated for each brain were used for the morphometric methods described later in this section. Group averaged DTI metric maps were also generated by averaging all DT volumes in a given group and then calculating the DTI metric values from the average tensor.

### Regions of Interest (ROI) Analysis

In order to consistently define individual native space ROI masks of particular DTI abnormalities, a semi-automated approach was designed to reduce user bias and to improve detection of subtle abnormalities by taking advantage of the mirror symmetry of brain hemispheres in this focal CCI model. First, “lateralized difference maps” were created for each specimen by DRTAMAS registration of the left-right flipped DTI volume to the original DTI volume followed by subtraction of the flipped FA and Trace maps from the original maps. The lateralized difference maps were then used in the ITKsnap semi-automated “snake tool” (Yushkevich et al., 2006) with a seed ROI placed in the region of abnormality and threshold values that were consistent values across all brains in the study. The snake-tool algorithm was then used to modify the seed region boundary based on the lateralized DTI map values and resulting in binary ROI masks for particular types of DTI abnormalities. Specifically, two types of focal abnormalities were used to generate separate masks: decreased Trace and decreased FA by generating thresholded “speed” images using FA_CCI_ - FA_contralateral_ < -0.5 and TR_CCI_ - TR_contralateral_ < -100. The seed growing region was constrained to the hemisphere ipsilateral to the CCI and the nature of the algorithm ensures that only regions of abnormal DTI values continuous with the seed region are included.

In order to visualize the extent and overlap of these masks in each group of injured brains, they were all warped to a common space and added within the group resulting in ROI overlay maps having integer values indicating the number of brains in that group with a ROI mask for each voxel. The volumes of the ROI masks were also calculated and used to quantify the lesion extent for each sample.

In order to compare the same tissue region across all brains, template-based ROI analysis was also performed by selecting a single ROI in template space and using it to extract DTI values from each individual brain map in template space. WM local to the injury site was investigated by creation of a single ROI mask in template space using the FA map template and a second ROI mask was also created for the corresponding region on the contralateral side. To investigate large bilateral WM structures, ROI masks were selected for the corpus callosum (CC), anterior commissure (AC), and internal capsule (IC) from existing ferret brain template masks.

### Voxelwise DTI Analysis

In order to automatically generate whole brain comparison maps to identify regions with abnormal DTI values in the CCI injured groups, voxelwise Cohen’s D maps for FA and Trace were generated according to DTI_Cohen’sD_ = (DTI_CCI_ - DTI_CON_)/SD(DTI_CON_) applied for each voxel.

### Conventional Tensor-Based Morphometry (TBM) and DTI-Driven TBM (D-TBM)

In order to detect local volume differences within the whole brain in an operator-independent manner, TBM methods (Davatzikos et al., 1996; Ashburner and Friston, 2000) were applied using both standard structural MRI-based TBM as well as a novel DTI-driven TBM (D-TBM) (Sadeghi et al., 2016; Nayak et al., 2017). TBM is a well-known MRI analysis approach that measures the determinant of the Jacobian (J) of the transformation to morph one brain volume to a template volume. A negative value of LogJ signifies that the individual brain is smaller than the template (i.e., atrophy or hypoplasia) while a positive value indicates the opposite. Log J values in this study were computed for each voxel from deformation field generated by the non-linear registration methods described above. For conventional TBM, these deformation fields were generated from registration of structural MRIs to the study specific average of all control brain volumes. Instead of using the T2-weighted images acquired in this study for TBM analysis, the amplitude images generated from DTI fitting were used to eliminate the potential effects from different pulse sequences. D-TBM in this study was performed using the deformation fields generated from the DRTAMAS registration described above of individual tensor volumes to the group averaged DT of the controls. D-TBM, which uses the registration of diffusion MRI data rather than anatomical images, takes advantage of the unique ability of diffusion MRI to depict individual WM pathways in regions that appear homogeneous in anatomical MRIs (Sadeghi et al., 2016). For both TBM and D-TBM, group LogJ maps were made by averaging the individual LogJ maps for all brains in each group.

### Statistical Analysis and Plots

Graphical and histogram plots were generated and statistical tests were performed to determine the significance of group differences FA, Trace, AD, RD, and LogJ values using the R statistical package^2^ (version 3.3.1). For individual ROI masks of the DTI abnormalities, volumes were plotted as points according to time after injury. For template-based ROI values from the WM of the injured gyrus and a comparable region on the contralateral side, a lattice of jittered scatterplots was made with group divisions according to time after injury. Within each plot, values from the contralateral and ipsilateral brain hemispheres within each sample were connected to show individual lateralized metric differences. Statistical testing of DTI metrics was performed using a two-way analysis of variance (ANOVA) with time after injury and side of injury as factors and the corresponding *F*-values and *p*-values are reported. For template-based ROI values from the CTX WM structures of the subcortical white matter (SC WM), and IC a lattice of jittered scatterplots were made for DTI values with group divisions according to time after injury and with the points from all ROIs included. ANOVA testing of LogJ and DTI values in the global WM was performed using a template-based WM mask.

Histogram analysis was performed using the R package “density” command to create density plots using group-averaged DTI metric maps and template-space ROI masks for the whole brain, WM, or CTX. Density plots were selected as way to compare the values and shapes of distributions for each group overlaid in the same plot. Consistent metric limits (shown on the *X*-axis of each density plot) and binning were used across groups to ensure unbiased comparisons.

### Histologic Staining and Immunohistochemistry

Histologic images from a subset of brains in this study were included for qualitative comparison with DTI abnormalities. To assess gross morphometric features of injury and to identify tissue regions with pathology, basic hematoxylin and eosin (H&E) staining (Richard-Allan Scientific Signature Series Hematoxylin 7211, ThermoFisher Scientific, Waltham, MA, United States) was performed using conventional methods. The slides were then scanned with a NanoZoomer 2.0-RS digital slide scanner (Hamamatsu Photonics K.K., Bridgewater, NJ, United States).

Immunohistochemistry was performed including staining for GFAP (1:500; Abcam, Cat#ab4674; RRID:AB_304558), IBA1 (1:1000; Wako, 019-19741; RRID:AB_839504), and MAP2 (1:100, Sigma–Aldrich, Cat#M4403; RRID:AB_477193). Images were acquired using a digital slide scanner at 10× magnification using Zen software (Axio Scan.Z1, Carl Zeiss Microscopy, Thornwood, NY, United States). Spatial correspondence of histologic and DTI abnormalities was determined using anatomic landmarks to identify a slice within the DTI volume that best corresponded to the histologic slice and then visually comparing whole slice and high magnification tissue features using standard tools in ImageJ^3^ software.

## Results

Mild CCI induced in several DTI and morphometric changes in the ferret brain including prominent focal abnormalities and subtle, widespread changes in DTI values and local volume. While all brains in this study were included for qualitative analysis, some of the injured brains (4/17) demonstrated more severe outcomes than expected according to surgical notes and MRI inspection and were excluded from quantitative analysis. The quality and resolution of diffusion MRI data in this study were sufficient for the visualization and quantitative analysis most major brain structures with smallest dimensions greater than 250 microns, which allowed WM measurements without the influence of partial volume effects.

### Focal DTI Abnormalities

Several prominent DTI abnormalities induced by the injury were detectable by visual inspection of the Trace and FA maps, including focal reductions of Trace primarily in the cortical tissue near to the CCI site and focally reduced FA of the WM in the same lobe of the CCI site.

#### Decreased Trace

**FIGURE 1:**
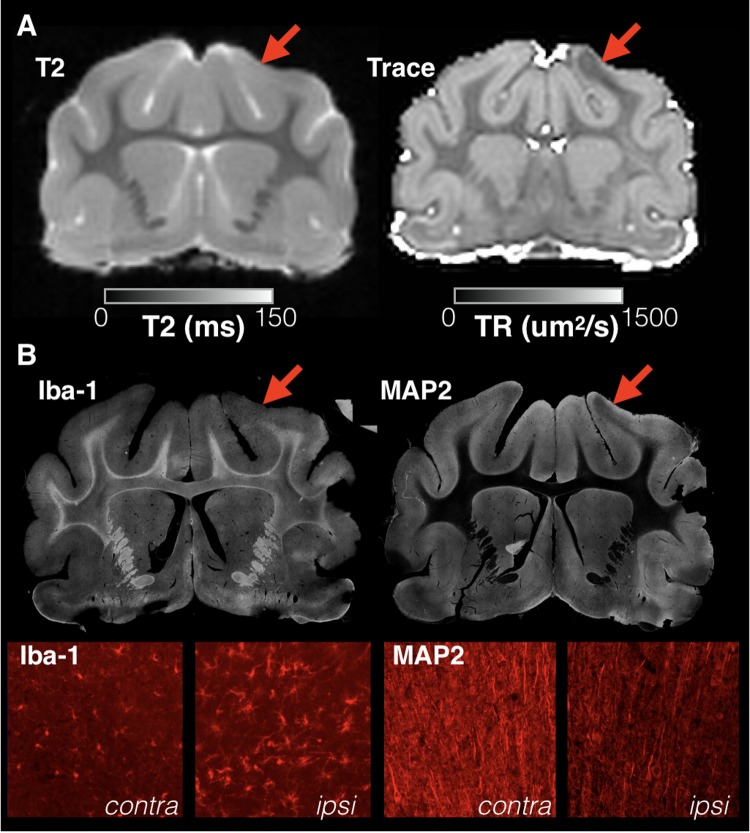
Focal and sharply delineated regions of decreased diffusivity were observed in the majority of injured brains in this study in the absence of overt T2 abnormalities. Whole slice images from the same brain specimen obtained 1 day following mCCI are shown at the same slice level for T2 and Trace MRI maps **(A)** as well as immunohistochemistry stained sections **(B)**. While T2 MRI of this brain was unremarkable, a prominent region of decreased Trace was found in the cortex near to the CCI site (red arrows) that colocalized with microgliosis revealed by Iba-1 staining and dendritic structural damage observed on MAP-2 staining. High magnification images are shown (bottom row) for both stains within the region of abnormal Trace (ipsi) and for the corresponding regions on the uninjured side (contra).

Of the injured brains included for quantitative analysis this study, 12/13 demonstrated a prominent region of low diffusivity that was visually evident near to the CCI site at all times after injury and generally was not accompanied by altered T2 values but did correspond with gliosis and abnormal neurite morphology (**Figure 1**). The average reduction in Trace across injured brains was 17% as compared with gray matter Trace values from the contralateral side.

#### Decreased Anisotropy

**FIGURE 2:**
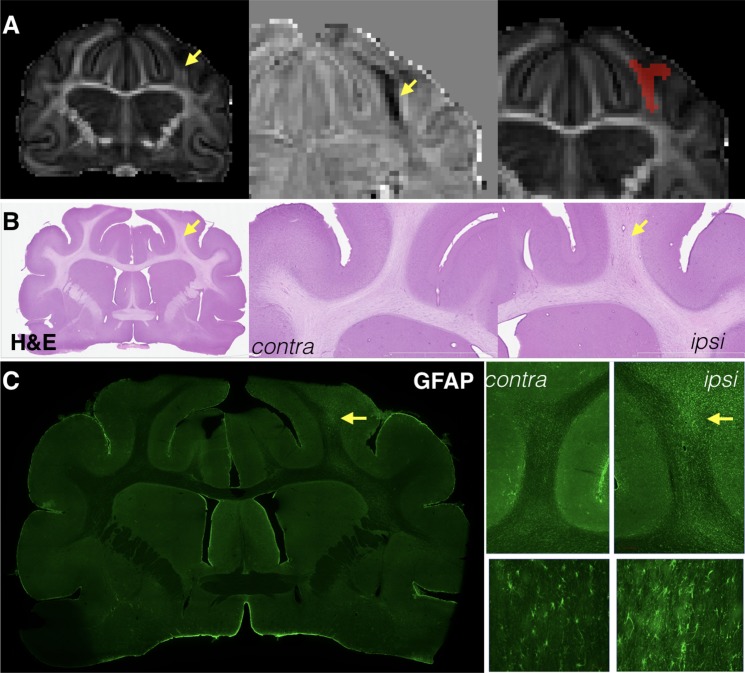
Focal reductions of fractional anisotropy (FA) were identified for most brains after mCCI and generally corresponded to pathology of the white matter near to the site of injury. Whole slice and magnified images are shown for the same representative brain taken 1 week after injury at the same slice level of the FA map **(A)**, H&E stained sections **(B)**, and GFAP immunohistochemistry **(C)**. Decreases in FA were in some cases visually observable **(A, left column)** and the shape of these abnormalities could be sharply defined by lateralized difference maps **(A, middle column)**, which were then used to generate ROI masks for this type of abnormality in a semi-automated way **(A, right)**. Cellular alterations in the same tissue region were subtle, but visually apparent by H&E staining **(B)** and more specifically found to involve astrocytosis by GFAP immunohistochemistry **(C)** in this specimen.

Changes in anisotropy were often, but not always, evident by eye in the affected lobe and 13/13 brains were found to have a region of reduced FA detectable using lateralized difference maps and corresponded with reactivity of the WM astrocytes and microglia (**Figure 2**).

#### Increased Trace

**FIGURE 3:**
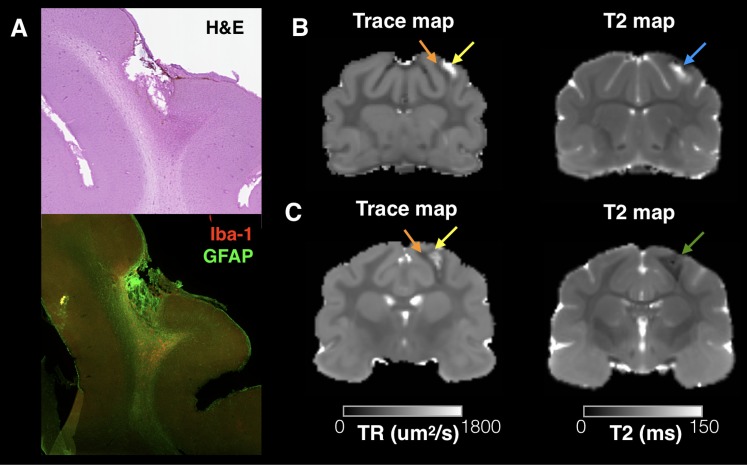
Trace and T2 abnormalities report different information about the tissue environment related to small focal cavitation. Regions of abnormally high T2 and Trace were observed in a subset of brains in this study that corresponded with small focal cavitations surrounded by glial scarring evident by H&E staining and IHC for Iba-1 and GFAP **(A)**. Examples are shown from two brains taken 4 and 12 weeks after CCI **(B,C)**, which demonstrate a consistent pattern of increased Trace in the cavity region (yellow arrows) and reduced trace in the surrounding region of glial scarring (orange arrows). T2 abnormalities were also abnormal near this pathology, but followed a different spatial pattern than Trace in each brain and with different T2 values across brains indicated by a blue arrow (**B**, increased T2) or green arrow (**C**, decreased T2).

The TBI model used in this study was meant to induce a mild injury without gross pathology, but a subset of brains demonstrated small focal cavitation surrounded by dense glial staining (see **Figure 3A**). The DTI associated with this pathology was a core region of increased Trace corresponding to the small cavity and an adjacent or surrounding region of decreased Trace corresponding to the dense glial staining. Notably, T2 values were also abnormal in these regions, although a clear correlation between Trace and T2 values was not observed (**Figures 3B,C**).

### ROI Overlay Maps and Lesion Volume Analysis

#### Decreased Trace

**FIGURE 4:**
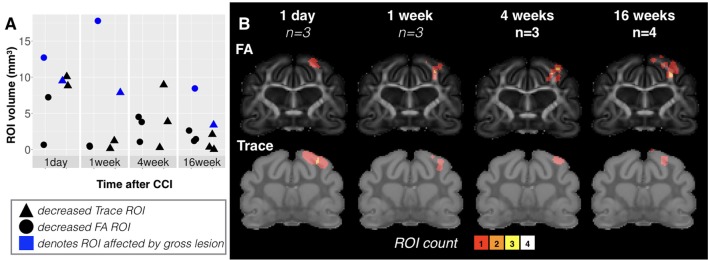
Volume, spatial extent and heterogeneity of focal DTI abnormalities at different times after mCCI. Volume values are plotted **(A)** for ROI masks generated based on Trace (triangles) and FA (circles) grouped according to time after injury. For values from brains with cavitation, points have been shaded in blue instead of black. Groupwise ROI overlay maps **(B)** combining all ROI masks within a group in a common space are shown to provide a visualization of the extent and heterogeneity of focal lesions at different times after mCCI. The sample size is given for each group and the color scale indicates the number of brains with a detectable abnormality at each voxel.

While Trace was prominently and reliably reduced in focal cortical regions, the spatial extent of this abnormality was heterogeneous across brains ranging in volume from under 1 mm^3^ to greater than 10 mm^3^. There was some dependence of lesion volume on time after CCI with all brains taken 1 day after injury having greater than 5 mm^3^ extent and those taken 16 weeks after injury having less than 5-mm^3^ extent (triangle points, **Figure 4**). The spatial pattern and localization of these regions of reduced Trace were notably heterogeneous across the brains of each group given that there was no voxel for which all brains in the same group demonstrated this abnormality when the brains were spatially coregistered (see ROI overlay maps of **Figure 4**).

#### Decreased FA

Unlike the Trace abnormality, reduced FA was most evident at later times after CCI and largely localized to the WM just below the CCI site – a region that was not directly impacted by the piston during the CCI procedure, but subject to secondary mechanical forces in the tissue such as pulling or shearing of the WM fibers. The spatial extent of reduced FA was variable at 1 day after CCI and included cortical tissue, but at later times after CCI, this abnormality became more consistently localized to the WM of the affected lobe.

### Template-Based Analysis of Focal DTI Abnormalities

**FIGURE 5:**
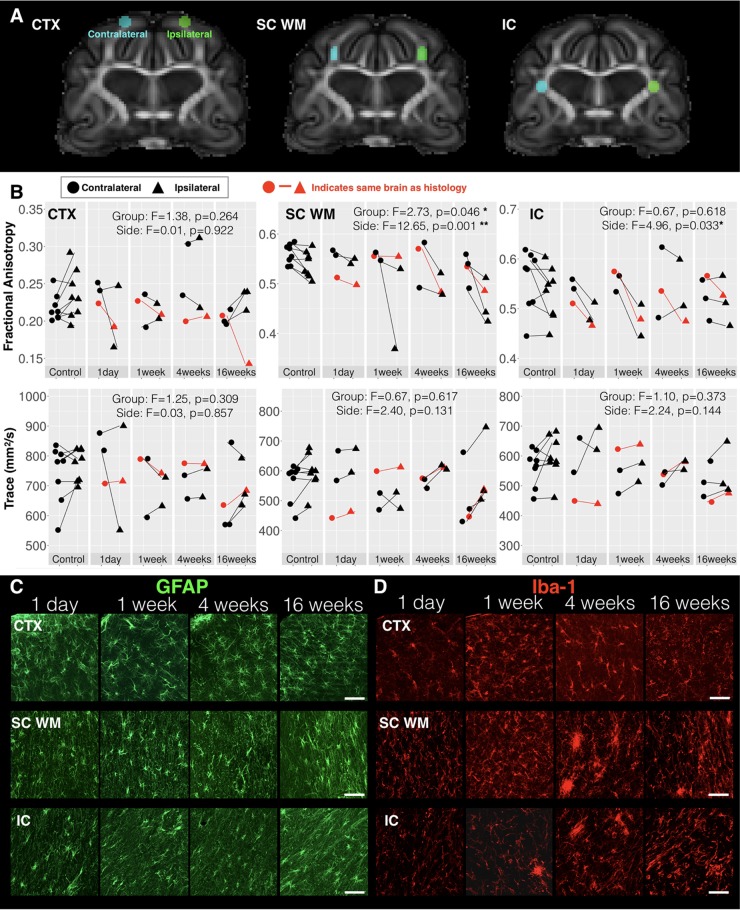
DTI values in regions of known histologic abnormality. Based on histologic observations following mCCI made by Schwerin et al. (2018), ROIs were placed on the *ex vivo* template **(A)** and used to extract FA and Trace values in the cortex (CTX), subcortical white matter (SC WM) and internal capsule (IC) corresponding to regions defined in the histologic study. Regional DTI values from the side of injury (triangular points) and those from the contralateral side (circular points) are plotted **(B)** for each time interval after CCI with lines connecting ipsilateral and contralateral values from individual samples and the results of two-way ANOVA with factors of side and time after CCI are given with asterisk to indicate significance levels (^∗^*p* < 0.05 and ^∗∗^*p* < 0.01). Fluorescent images of immunohistochemistry stained sections from ipsilateral CTX, SC WM, and IC regions of brains from different times after injury are shown for GFAP staining of astrocytes **(C)** and Iba-1 staining of microglia **(D)**. The brain specimens for each time point that were used for histology are indicated by red lines in B and the white scale bar indicates 50 microns.

The DTI values were evaluated in the CTX, SC WM, and in a focal region of the IC using template-defined ROIs placed according to previous histological findings (Schwerin et al., 2018). Extracted FA and Trace values for each brain in template space are shown in **Figure 5** according to time after CCI. While FA values on the uninjured side were relatively constant across groups, FA values on the injured side were decreased compared with the uninjured side at later time points. No significant differences were detected for the CTX, but in the SC WM two-way ANOVA with factors of time after CCI and brain hemisphere found a significant main effect for both group (*F* = 2.73, df = 4, *p* = 0.046) and hemisphere of injury (*F* = 12.65, df = 1, *p* = 0.0012) for FA and a main effect of hemisphere for RD (*F* = 6.99, df = 1, *p* = 0.013). In the local IC, a main effect of hemisphere was found for FA (*F* = 4.96, df = 1, *p* = 0.033) and also for RD (*F* = 4.67, df = 1, *p* = 0.038). Neither the SC WM nor the local IC were any significant effects of group or side found for Trace or RD. Targeted histologic images were acquired from the same brains used for imaging to provide qualitative characterization of astrocytes and microglia in the same region as the ROIs. Immunoreactivity for the three WM regions studied for FA and Trace analysis showed altered staining patterns for GFAP and Iba-1 reactivity (**Figure 5C**). Although these glial markers exhibited changes from 1-week post injury not strongly evident in the FA data, there were strong immunoreactivity modifications that correlated with the FA alterations seen in WM regions, especially at our longer survival times (also see Schwerin et al., 2018). These were most consistent with the temporal dependence of FA changes in the same brains (red lines, **Figure 5B**). The cerebral CTX also demonstrated positive GFAP and Iba-1 staining, but with less evident morphologic alterations and less dependence on time after CCI.

### Voxelwise Subtraction Maps Show Focal and Global DTI Abnormalities With Temporal Dependence

**FIGURE 6:**
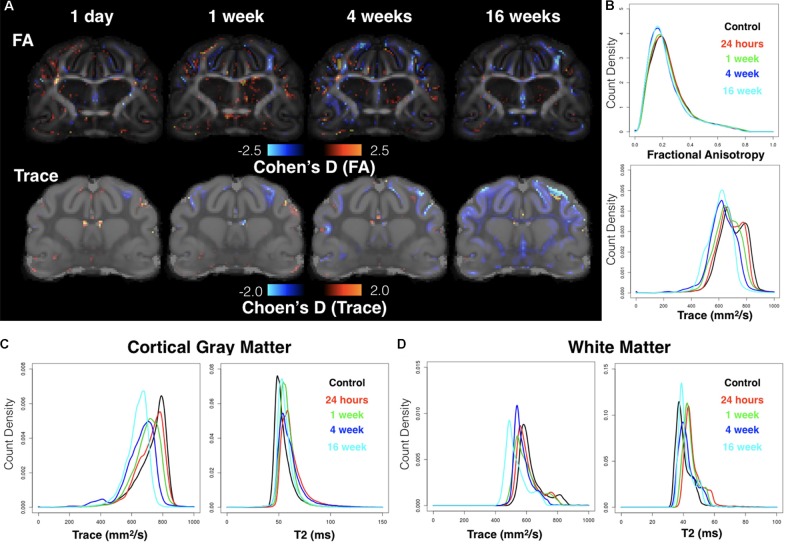
Whole-brain analysis using voxelwise effect size maps and DTI metric histograms. Cohen’s D maps for FA and Trace are shown at the same slice level for each group according to time after CCI **(A)** where voxels with increased or decreased DTI values as compared with the control group are indicated by warm and cool colors respectively. Whole-brain histogram analysis **(B)** of FA and Trace values from the average template brain maps for each group are shown as well as Trace and T2 histograms for cortical gray matter **(C)** and whole brain white matter **(D)**.

Cohen’s D maps for Trace revealed both focal abnormalities especially for the acute time point near to the CCI site and a widespread pattern of reduced Trace during the chronic period that was quite distinct from the focal observations. Whole brain histogram analysis of Trace (**Figure 6B**) confirmed these observations showing a leftward shift of Trace values depending on time after mCCI. Because this result was not expected, additional histogram analyses of Trace and T2 (**Figures 6C,D**) were performed to determine if factors related directly to the MRI signal – and possibly due to differences in sample preparation or image acquisition – could be related to the observed shift in Trace. In the control group, T2 histograms were similar across brain specimens with the exception of two brains with a large shift to increased T2 values and decreased Trace values. In contrast to the histogram behavior for these two control brains, individual histograms of injured brains that were found to have globally decreased Trace values did not demonstrate shifts in T2 values. Group averaged histograms in the cortical gray matter (**Figure 6C**) and in the global WM (**Figure 6D**) showed selective shifts in Trace, but not T2.

Reduced FA was evident from Cohen’s D maps (**Figure 6A**) with a modest reduction of FA during the acute period and more prominent decreases in FA evident during the chronic period that were persistent at 16 weeks and involved the WM. There was no evidence of widespread alterations of FA values as were found for Trace.

### Template-Based Analysis of Large Anatomic Structures Shows Reduced Chronic Stage White Matter Diffusivity

**FIGURE 7:**
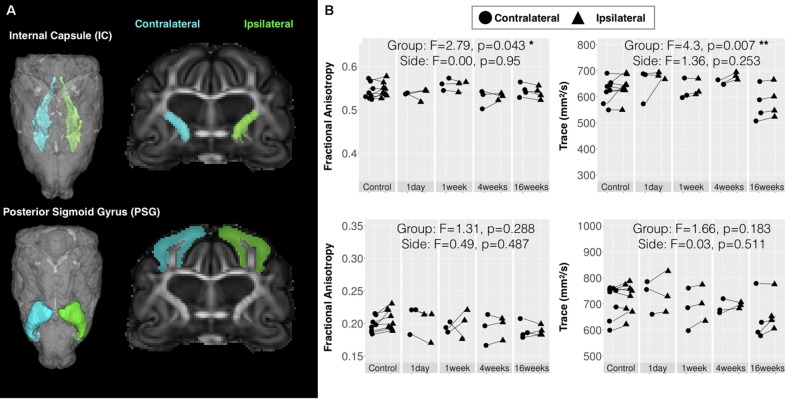
Quantitative template-based analysis of DTI values in large bilateral regions. Template ROI masks for the internal capsule (IC) and posterior sigmoid gyrus (PSG), which was target for CCI are shown **(A)** in the 3D rendered whole brain view and in a coronal slice for the ipsilateral (green) and contralateral (cyan) side. **(B)** DTI values extracted from each specimen in these ROIs are plotted for each according to time after injury where ipsilateral values (triangular points) are connected to contralateral values (circular points) of the same brain by lines and the results of two-way ANOVA with factors of side and time after CCI are given with asterisk to indicate significance levels (^∗^*p* < 0.05 and ^∗∗^*p* < 0.01).

Two brain regions were selected from a set of existing ferret template ROI masks (Hutchinson et al., 2017b) based on their potential relevance to the injury in this study and DTI values were extracted and compared across according to time after injury and side relative to CCI site (**Figure 7**). The posterior sigmoid gyrus (PSG) – the cortical region that was the target of the CCI injury in this study – was not found to have significant alteration of DTI values, which suggests that the changes induced by mild CCI that can be detected by DTI are not extensive within the PSG. The full length of the IC was also investigated based on the report of gliosis in a small region of this structure. No effect of side were found for DTI measures in this region, but significant main effects of time after injury for FA (*F* = 2.79, df = 4, *p* = 0.043), TR (*F* = 4.30, df = 4, *p* = 0.007), AD (*F* = 3.16, df = 4, *p* = 0.027), and RD (*F* = 5.46, df = 4, *p* = 0.002) were found for this large WM structure that were more significant for Trace than FA. Plots of these demonstrated a bilateral reduction of diffusivity in this region during the chronic period.

### Morphometric Changes in the White Matter During the Chronic Phase Using D-TBM Analysis

**FIGURE 8:**
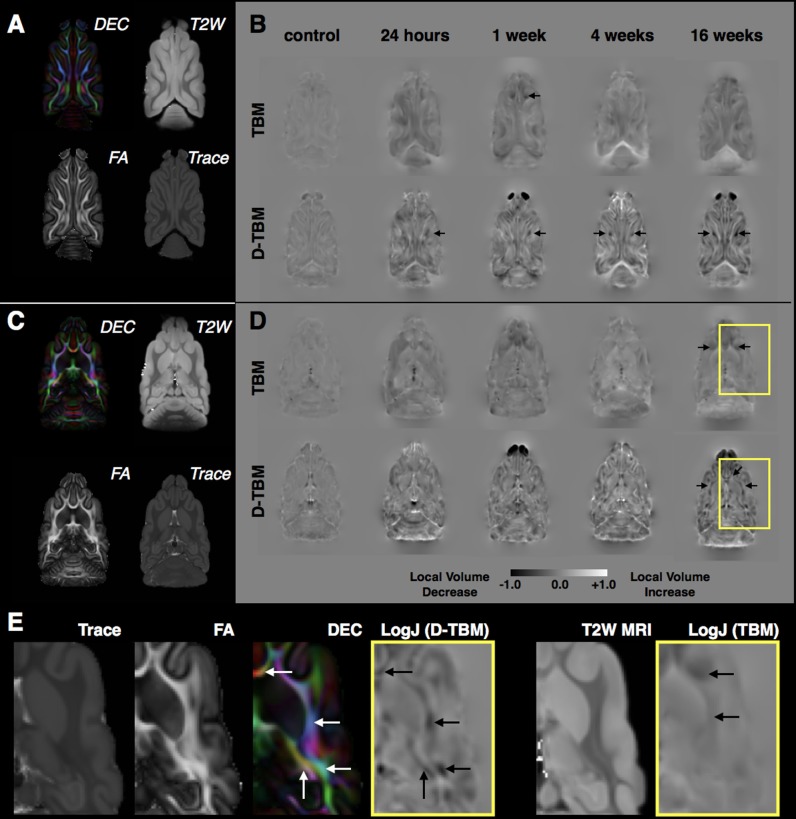
Tensor-based morphometry analysis of local volume changes following mCCI. For both a dorsal slice near to the CCI site **(A,B)** and a more ventral slice distant from the CCI site **(C,D)**, DTI maps from the control template are shown for reference **(A,C)** and group averaged LogJ maps generated from conventional TBM using structural MRI registration and D-TBM using DTI-based registration are shown for each experimental group **(B,D)** with the same window scaling as indicated in the scale bar at bottom. Black arrows indicate regions of reduced volume, which are more apparent by D-TBM analysis and most prominent near to the CCI site during the acute period but found to be bilateral and widespread during the chronic period. Closer examination of D-TBM LogJ maps from 16 weeks after mCCI **(E)** reveals several regions of white matter (WM) volume reduction (black arrows, D-TBM) that are localized to particular tracts within the lager body of WM discernable by their orientation on DEC maps (white arrows) compared with homogeneous contrast on scalar maps (Trace, FA, and T2W MRI) resulting in less focal or absent conventional TBM findings (black arrows, TBM).

Local volume changes were detected following mCCI using D-TBM that was distinct from conventional TBM results. LogJ maps from both approaches (**Figure 8**) reported voxelwise reductions (negative or darker values) and increases (positive LogJ or brighter values) of volume in each group indicating preferential volume reductions in WM regions and increases in ventricle regions. As well, the pattern of changes across groups appeared to be dependent on the time following injury with ipsilateral abnormalities apparent in the acute period and bilateral and widespread volume changes evident during the chronic period. While both conventional TBM and D-TBM LogJ maps shared these general features, the D-TBM approach appeared to provide more detailed information with sharper contrast in smaller regions, especially of WM tracts within larger WM tissue regions. D-TBM findings showed reduced LogJ values near the CCI site within the first week after injury, but more widespread and bilateral changes during the chronic period that were most pronounced at 16 weeks after mCCI. A main effect of time after injury was found for LogJ values (*F* = 4.32, df = 4, *p* = 0.007), but not for DTI values in the whole brain WM.

## Discussion

In a model of mTBI in the ferret, we found DTI abnormalities that ranged from prominent focal changes to subtle widespread alterations including changes that were undetectable with relaxometry-based MRI. The observed DTI abnormalities were consistent with the time course and spatial patterns of hallmark mTBI pathologies such as DAI, inflammation and WM vulnerability. As well, morphometric analysis by D-TBM revealed progressive and widespread atrophy of WM structures distant from the primary lesion. The work presented here extends and refines what has been shown in rodent models and recapitulates several notable DTI and morphometric findings from human studies of mTBI.

### Spatio-Temporal DTI and Morphometric Alterations Following mTBI

Using high-resolution voxelwise analysis, focal DTI abnormalities were observed for each injured brain and found to be dependent on the time interval after injury. Trace and FA were reduced 1 day after CCI in tissue regions directly impacted by the CCI device and in adjacent tissue. At later times after CCI, regions of reduced Trace were less extensive, but FA reductions became apparent in the underlying WM. For most of the observed focal DTI abnormalities there was no accompanying T2-MRI abnormality and for the subset of brains demonstrating altered T2, there was no direct correspondence between T2 abnormalities, DTI metrics, and cellular alterations. Taken together, this demonstrates the ability of DTI to consistently and sensitively detect regions of tissue alteration invisible to conventional MRI in this model.

In addition to focal injury, global changes in diffusivity to lower values were observed that appeared to be time dependent. This finding was highly evident in effect size maps for Trace and on subsequent analysis by histogram comparisons, which indicated a global reduction in diffusivity that was most pronounced for brains observed 4–16 weeks after CCI. Secondary analysis by template-ROI methods demonstrated that these changes were structure-specific with large bilateral WM structures having different temporal profiles, possibly from the spread of diffuse changes. While reduced diffusivity that is widespread can arise from TBI-related cellular alterations (e.g., chronic global inflammation and WM gliosis), they may also relate to experimental factors or artifacts.

Recent histological analysis of the brains analyzed by DTI in the present study demonstrated a progressive pattern of reactive microglia and astrocytes emerging following CCI with pronounced cortical gliosis during the acute stage followed by the appearance of reactive glia – both astrocytes and microglia – in the WM during the chronic stage (Schwerin et al., 2018). Focal template-based ROI analysis of Trace and FA values in these same brains and regions revealed consistent radiologic-pathologic relationship within the WM for which significant differences were observed in both the SC WM and in a focal region of the IC. While the SC WM demonstrated a pattern of decreased FA that was most robust in the chronic period and thus consistent with glial reactivity outcomes, the DTI profile of reduced FA at 1 day and 1 week after CCI in the IC was not fully consistent with the histologic findings. This may be related to a limitation of the sensitivity of DTI to detect lesser levels of pathology or to a more complex neurobiological environment in the IC than in the SC WM. Nevertheless, the similarities in spatio-temporal observations of abnormalities by both DTI and histology suggest a preferential vulnerability of the WM following CCI and a relationship between FA and glial changes.

The final major finding of this study was subtle but detectable atrophy of particular WM tracts using the D-TBM approach. Selective focal reductions in WM volume were found in the acute phase in the WM regions near to the CCI site, but by 16 weeks after injury, bilateral volume reductions were evident in a number of WM regions distant from the site of injury. Notably, these morphometric abnormalities were evident only by D-TBM but were not detectable by conventional TBM. This implies that tensor-based registration methods (Irfanoglu et al., 2016) which are sensitive to local tissue geometry within larger tissue regions (e.g., WM) may be advantageous for detecting subtle atrophy following mTBI.

### Study of mTBI in the Ferret Extends What Has Been Learned From Rodent Models

The finding of focal reductions of *ex vivo* diffusivity near to the CCI site during the acute phase contributes to a long history of diffusion MRI observations from rodent models of TBI, in which both increases and decreases in diffusivity were reported in the earliest studies (Hanstock et al., 1994; Alsop et al., 1996) and the prescient observation was made (Smith et al., 1995) that the divergence in diffusivity findings is likely due to severity. Mechanistically, this is supported by studies of acute ischemia showing diffusivity reduction associated with edema (Moseley et al., 1990) that persists even when cell suffering is severe but cell membranes are still intact (Pierpaoli et al., 1993). However, in non-ischemic regions and in regions with mild cellular impairment, the presence of interstitial edema results in increased water diffusivity (Pierpaoli et al., 1993). In this context, the findings of the current study – reduced diffusivity corresponding to mild cellular damage – seem at first in contrast with these previous finding in ischemia. However, it is important to consider potential differences between *in vivo* and *ex vivo* assessment. It is well known that the interstitial space is reduced with perfusion fixation (Cragg, 1979; Korogod et al., 2015) such that the effect of interstitial edema may not be evident in *ex vivo* diffusion MRI acquisitions. In fact, an initial *in vivo* study of a subset of ferrets from the current work (Hutchinson et al., 2016) found that diffusivity is increased after mTBI in the ferret, but when the same brain is imaged *ex vivo*, diffusivity is reduced in the affected region. Thus, cellular alterations from mild damage (e.g., cellularity, beading, gliosis) may be present following mTBI, but masked by the overwhelming influence of interstitial edema *in vivo* and then unmasked when performing *ex vivo* DTI. This point underscores the value of more advanced diffusion modeling for TBI research (Hutchinson et al., 2017a) that could potentially separate the relative contributions of edema and cellular alterations to abnormal diffusivity *in vivo*.

Reduced FA in regions of WM, such as those observed in the present study, are a nearly ubiquitous finding in rodent models of TBI that consider them (Mac Donald et al., 2007a,b; Bennett et al., 2012). However, the small dimensions of WM in the rodent have always presented the potential confound of partial volume effects whereby reduced FA could be driven by reduced relative volume of WM within a voxel instead of altered WM microstructure. Findings of reduced focal WM FA from the present study in ferrets, which do not suffer from partial volume effects in WM, support a microstructural interpretation – at least in part – of earlier work in rodent models. Furthermore, FA reductions were accompanied by increased radial diffusivity, which supports similar findings in rodent during the chronic period following CCI (Budde et al., 2011; Li et al., 2011).

An interesting discrepancy between observations from the present study and previous studies from rodents (Budde et al., 2011) including our own observations in mice (Hutchinson et al., 2014) is the lack of prominently increased FA near to the CCI site in the ferret. This could perhaps be due to the lower relative volume of CCI impacted tissue for the ferret or could arise from differences in underlying geometric complexity of the ferret and mouse cortical tissue. Additionally, increased cortical FA has not yet been described in human studies, so it is important to understand the potential interpretation of this abnormality in rodents and the extent to which it is useful as a pre-clinical outcome measure.

While CCI in the ferret was found to provide an important basic model of mild focal injury with detectable imaging abnormalities, it is also important to point out that these post-TBI outcomes may be different in models that recapitulate diffuse features more in common with human mTBI such as blast or closed head injury. As well, repetitive mild TBI may have additional or different imaging outcomes from single TBI paradigms. The combination of such translationally relevant TBI models with selection of a species such as the ferret with brain features in common with humans may improve the utility of pre-clinical studies as a way to understand human TBI and to develop clinically meaningful MRI markers.

### Similarity of mTBI Outcomes Between the Human and Ferret

A primary goal for neuroimaging in this ferret model of mTBI was to explore the similarity of imaging findings with those that have been described in humans as a way to bridge pre-clinical and clinical studies and develop meaningful diagnostic imaging markers. MRI and DTI studies of mTBI in humans have identified a range of potential markers (Haacke et al., 2010; Shenton et al., 2012; Hulkower et al., 2013), many of which are recapitulated here including focal FA reductions, diffuse WM DTI abnormalities and WM atrophy. Focal DTI alterations were found in regions of confirmed axonal injury and gliosis near to the site of mild impact which is consistent with findings in human mTBI of localized FA reductions hypothesized to correspond to axonal injury (Arfanakis et al., 2002). While the radiologic-pathologic correspondence observed here should also be examined in future human brain studies, these findings suggest that astrocytosis and microglial clusters underlie progressive FA decreases in WM after mTBI and may also explain similar observations in human TBI. However, the small spatial extent, heterogeneity of spatial localization across individuals and temporal dependence of focal abnormalities in this study underscore the current limitations of DTI for detecting subtle focal pathology (Ilvesmaki et al., 2014) and the methodological caveats that have been pointed out for using DTI in studies of mTBI (Delouche et al., 2016).

The combined results of widespread Trace reduction in large bilateral WM regions and D-TBM evidence for WM atrophy suggest that mild CCI in the ferret results in late appearing abnormalities that are selective to WM regions and distant from the injury site. This may provide a set of important outcome measures for the development of therapeutic intervention during the chronic period. The detection of diffuse microstructural abnormalities may be more robust across injury types (i.e., focal and non-focal) and several DTI studies in humans have reported similar WM changes (Benson et al., 2007; Yuh et al., 2014) and volumetric studies in human mTBI that show preferential vulnerability of the large WM tracts (Ding et al., 2008). Conventional TBM studies (Kim et al., 2008; Sidaros et al., 2009; Farbota et al., 2012) in moderate and severe TBI have also found selective WM atrophy in the chronic phase. Notably, conventional TBM in the present study did not capture WM atrophy with the same level of detail as the DTBM approach, likely because DTI-based registration improves local tract alignment and consequently DTBM provides greater sensitivity to tract-specific volume changes within regions of multiple WM tracts that cannot be discerned with anatomical images. D-TBM should be considered as a powerful technique to be employed in human studies to monitor delayed WM damage following TBI.

## Conclusion

The mTBI research lacks tools that are sensitive to the subtle alterations that follow injury and pre-clinical models that are able to adequately mimic human outcomes. This study has performed advanced diffusion tensor MRI (DTI) analysis in a human-similar model of TBI in the ferret in order to identify promising imaging markers of brain tissue alterations that are relevant for advancing basic and translational research of mild TBI. A set of imaging abnormalities were identified and characterized in this study with a range of spatial and temporal features that extend previous observations in rodent models and are related to key aspects of human TBI including WM vulnerability and chronic microstructural and morphometric abnormalities detectable by DTI.

## Ethics Statement

This study was carried out in accordance with the recommendations of national guidelines (the Guide for the Care and Use of Laboratory Animals) and institutional oversight by the Uniformed Services University of Health Sciences Animal Care and Use Committee.

## Author Contributions

EH, SS, SJ, and CP performed the experimental design. EH and MK performed the MRI acquisition. EH, NS, and MI performed the processing and analysis. SS, KR, and SJ performed the histopathology processing, analysis, and figure preparations. EH wrote the manuscript with the substantial editing input from CP. All the authors contributed to the research that was described in this manuscript.

## Conflict of Interest Statement

The authors declare that the research was conducted in the absence of any commercial or financial relationships that could be construed as a potential conflict of interest.

## Footnotes

1 http://www.itksnap.org

2 www.r-project.org

3 https://imagej.nih.gov/ij/
